# Experimental Study and Regression Modeling of Sound Absorption Coefficient for Wood Panels

**DOI:** 10.3390/ma18245488

**Published:** 2025-12-05

**Authors:** Miljenko Krhen, Marin Hasan, Franjo Bolkovac, Kristijan Radmanović

**Affiliations:** 1 University of Zagreb Faculty of Textile Technology, Prilaz baruna Filipovića 28a, 10000 Zagreb, Croatia; 2 University of Zagreb Faculty of Forestry and Wood Technology, Svetošimunska cesta 23, 10000 Zagreb, Croatia; mhasan@sumfak.unizg.hr (M.H.); kradmanovic@sumfak.unizg.hr (K.R.); 3 Franakustik Ltd., Cesargradska ulica 3a, 10000 Zagreb, Croatia; fbolkovac@gmail.com

**Keywords:** sound absorption coefficient, perforated wooden panels, wood density, panel thickness, perforation ratio, impedance tube, regression model, experimental acoustics

## Abstract

This study presents a predictive model for estimating the sound absorption coefficient of perforated and non-perforated wooden panels, based on experimental data. Measurements were conducted on four wood species: fir wood (*Abies alba*), pine wood (*Pinus sylvestris*), pedunculate oak (*Quercus robur*), and sessile oak (*Quercus petraea*) in three panel thicknesses (11 mm, 18 mm and 25 mm), with perforation ratios of 0%, 10%, and 20%. The normal-incidence absorption coefficient was measured using the impedance tube method in accordance with ISO 10534-2. Measurements were performed in a 100 mm impedance tube, selected to match the specimen dimensions; therefore, the analysis is limited to the valid plane-wave frequency range of this tube, between 250 and 1600 Hz. Previous studies have shown that both panel thickness and perforation ratio significantly influence mid- and high-frequency absorption. Our results confirm that increased panel thickness and perforation enhance absorption, consistent with findings reported for micro-perforated and porous wood panels. Based on the measured values, we developed first-order regression functions linking the absorption coefficient to material density, thickness, and perforation percentage. The resulting equations allow reverse estimation of one or more physical parameters to meet target acoustic performance requirements. This data-driven approach provides a practical tool for designing wooden absorbers with predictable behavior and complements existing analytical models for acoustic optimization.

## 1. Introduction

Architectural acoustic design often requires balancing aesthetic considerations with strict acoustic performance targets. In modern interior architecture, especially in auditoriums, classrooms, public halls, and corporate environments, there is increasing demand for wooden panels as acoustic finishing elements. These panels are frequently specified to match a visual identity or design concept proposed by architects, which can limit the acoustic engineer’s flexibility to select optimal material parameters. In this context, a methodology that enables reverse prediction of the absorption coefficient or the required material configuration is not only desirable but necessary.

Wooden panels, both solid and perforated, are widely used due to their sustainability, versatility, and visual warmth. However, their acoustic performance varies significantly depending on material parameters such as thickness, density, and perforation ratio. In many practical applications, the designer must start not with a set of known materials but with a target absorption coefficient derived from the room acoustic design process. From this starting point, the goal is to identify the most suitable combination of thickness, density, and perforation that meets both the acoustic and structural requirements of the project.

This study addresses this challenge by providing a practical method for estimating the sound absorption coefficient of wooden panels based on three key physical parameters: wood density, panel thickness, and perforation percentage. Furthermore, it enables reverse calculation to determine the necessary thickness and/or perforation level required to achieve a specific absorption coefficient at selected frequency bands. This approach is critically important in architectural projects where high surface coverage of acoustic panels must be achieved without excessive structural loads. In such cases, selecting a lower-density wood species or reducing thickness while compensating with perforation can achieve the required performance with lower mass.

In architectural practice, panels are typically designed before the final acoustic simulation stage. This sequence often leads to compromises between appearance and performance, especially when solid or low-perforation designs are preferred for visual reasons. Unfortunately, these designs may underperform acoustically, particularly at mid and high frequencies. Conversely, heavily perforated panels or thicker boards with improved absorption may not align with the visual intent or construction constraints of the design. Therefore, tools that enable prediction and material tuning based on fixed design constraints are essential.

This research is based on systematic measurements of sound absorption [1]. The resulting dataset enables the construction of first-order regression functions relating to the normal-incidence sound absorption coefficient to each of the three physical variables.

The practical benefit of these regression functions lies in their bidirectional utility. They enable forward prediction: if the wood type (and thus density), thickness, and perforation ratio are specified, the designer can estimate the expected absorption coefficient. More importantly for architectural integration, these functions support reverse engineering: given a desired absorption coefficient at one or more frequencies, one can calculate the appropriate combination of thickness, density, and perforation ratio to achieve that performance.

Moreover, these parameters are interdependent not only in acoustic performance but also in structural behavior. The total surface mass of the installed panels affects the load-bearing capacity of ceilings and partition systems. This is especially critical when retrofitting heritage buildings or designing lightweight modular interiors. The ability to reduce panel mass by optimizing thickness and density, without sacrificing acoustic performance, is a key advantage offered by the presented model.

This work complements prior studies in the field, such as those investigating micro-perforated panels [2], artificial neural network predictions [3], and finite element simulations [4], while focusing on empirical regression models that are directly applicable in design workflows. By providing a tool to bridge the gap between aesthetic constraints and acoustic requirements, this research contributes to more integrated and efficient acoustic design processes.

From a research perspective, the acoustic behavior of wood-based absorbers has been investigated in several complementary directions. Experimental and theoretical studies on perforated wooden panels and plywood have clarified the influence of perforation geometry, panel thickness and backing cavity on the absorption spectrum [4,5]. Other works have focused on micro-perforated or micro-grooved elements and on wood-fiber, bark and wood-chip absorbers in order to achieve broadband absorption using primarily wood-based or bio-based materials [6]. Data-driven and machine-learning approaches, such as artificial neural networks, have also been applied to estimate the absorption of perforated wooden panels [3]. However, these contributions typically address specific engineered products, narrow ranges of geometrical parameters, or individual wood species, and they often result in models that are not readily translated into simple design rules for everyday architectural practice.

Consequently, there remains a practical research gap for solid and perforated wooden panels that are selected primarily for aesthetic reasons but must also satisfy well-defined acoustic targets. Designers still lack compact, transparent relationships that directly link basic material parameters—wood density, panel thickness and perforation ratio—to the sound absorption coefficient across several commonly used wood species. The present study addresses this gap by providing a systematic experimental dataset obtained from impedance tube measurements on four wood species and by deriving first-order regression functions that relate the normal-incidence absorption coefficient to density, thickness and perforation percentage. These relationships are intentionally kept simple so that they can be used as practical tools for forward prediction and inverse design when selecting materials and geometries for the acoustic treatment of wooden panels in buildings.

## 2. Materials and Methods

### Specimen Fabrication for Acoustic Measurements

All specimens used in this study were made from kiln-dried solid wood boards to ensure dimensional stability and consistent material properties. The fabrication process was carefully designed and implemented as a unique, purpose-built production sequence developed exclusively for this acoustic investigation. This custom fabrication approach allowed strict control over critical variables such as wood species, panel thickness, perforation geometry, and surface finish.

From each selected wood species, raw planks with uniform grain and no visible surface defects were first dried to a moisture content of 8–10%. They were then planed and cut into flat boards of three standardized thicknesses: 11 mm, 18 mm and 25 mm. Circular specimens were machined from these boards using a custom jig to match the internal diameter of the impedance tube used in testing, thus ensuring a precise fit and repeatability across all measurements. Each disk was precisely dimensioned using a high tolerance cutting system to eliminate edge gaps that could affect measurement accuracy.

**Figure 1:**
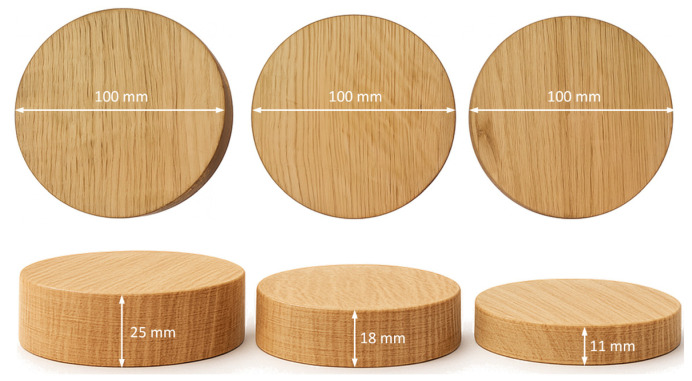
Prepared non-perforated Sessile oak specimens.

Circular specimens with a diameter of 100 mm were cut from these boards using a computer numerical control (CNC) milling machine to match the inner diameter of the impedance tube used in the measurements, thereby ensuring a perfect fit of the test specimen inside the tube. Using this procedure, ten specimens of each wood type were produced, each with a diameter of 100 mm and thicknesses of 11 mm, 18 mm, and 25 mm. Figure 1 shows the prepared specimens of Sessile Oak.

**Figure 2:**
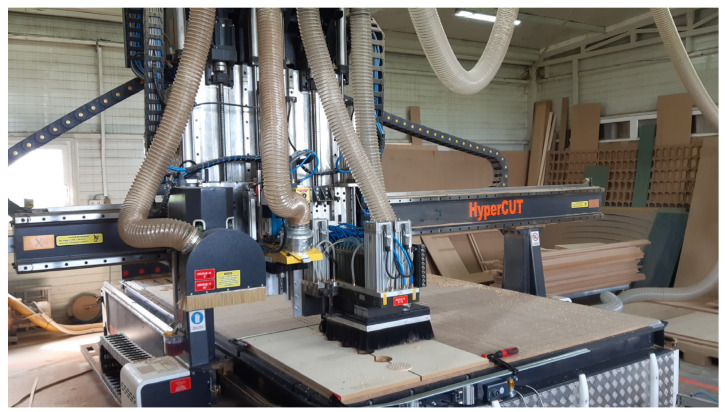
CNC milling machine used to fabricate wooden test specimens. A custom clamping mask ensured repeatable alignment and consistent perforation geometry across all specimens.

Perforations were subsequently introduced on selected specimens using a CNC milling machine (CNC HyperCUT 2030 AMO PRO, Gorišnica, Slovenia), as shown in Figure 2. CNC technology enabled precise control of hole diameter, spacing, and layout pattern, ensuring consistency between replicates and across different material types. One group of specimens remained unperforated (0%) to provide a baseline for comparison.

**Figure 3:**
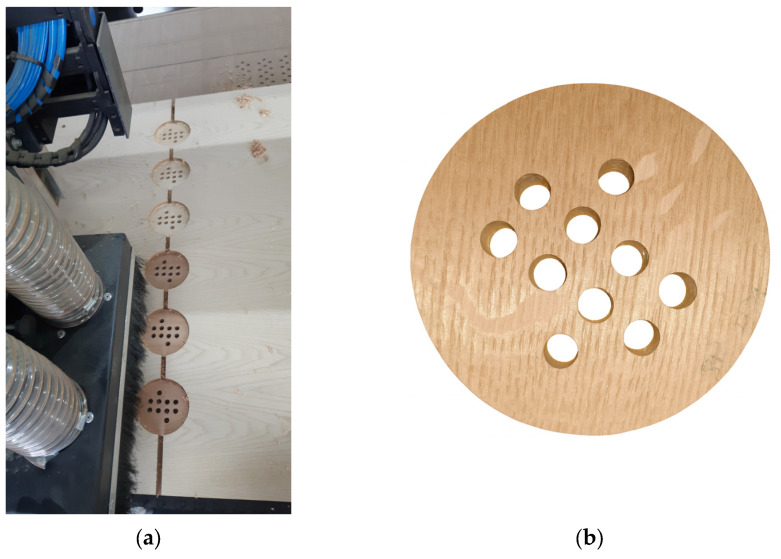
Wooden test specimens prepared for impedance tube measurements. (**a**) Specimen fabrication on a CNC machine using specially prepared templates; (**b**) specimen with 10% perforation ratio.

All perforated patterns used a uniform grid layout to simulate common architectural perforation schemes while minimizing diffraction and boundary effects. To maintain consistent airflow resistance characteristics, all holes were drilled cleanly through the panel thickness without countersinking or surface tapering. Each pattern was optimized in CAD software (Aspire ver. 9.5, Vectric Ltd., Alcester, UK) before fabrication and validated against theoretical open area calculations. Specially prepared templates and the specimen with a 10% perforation ratio are shown in Figure 3.

All fabricated specimens were individually inspected, labeled, and stored under controlled environmental conditions before measurement. Care was taken to ensure that each specimen represented the target combination of parameters and conformed to guidelines for impedance tube testing. The complete set of specimens, including four wood species, three thickness levels, and three perforation conditions, provided a comprehensive matrix for evaluating the effects of key physical variables on the sound absorption coefficient.

Two perforation configurations were implemented: p_1_ = 10% and p_2_ = 20%, corresponding to the ratio of the total open area to the total area of the front surface of the disc, as defined in (1), where *p* is the perforation ratio, *N* is the number of perforated holes, *r* is the radius of an individual perforation (*r* = 5 mm), and *R* is the radius of the front surface of the disc (*R* = 50 mm). Using (1) and the specified perforation ratios p_1_ = 10% and p_2_ = 20%, the corresponding numbers of perforated holes were determined as N_1_ = 10 and N_2_ = 20.

(1) $$p=\frac{N\cdot r^{2}}{R^{2}}$$

Prior to measuring the sound absorption coefficient, each specimen was individually weighed, and its density was determined according to Expressions (2)–(4), where m_0_ is the mass of the non-perforated specimens, V_0_ is the volume of the non-perforated specimens, m_1_ is the mass of the specimens at *p*_1_ = 10%, and m_2_ is the mass of the specimens at *p*_2_ = 20%. In the acoustics of perforated panels, it is standard to define perforation as the percentage of open surface area. Since all specimens were perforated through their full thickness, the volumetric perforation is equal to the surface perforation, and the above expressions can therefore be applied.

(2) $$\rho_{0}=\frac{m_{0}}{V_{0}}$$

(3) $$\rho_{1}=\frac{m_{1}}{0.9\cdot V_{0}}$$

(4) $$\rho_{2}=\frac{m_{2}}{0.8\cdot V_{0}}$$

## 3. Parameter Extraction from Measured Absorption Data

**Figure 4:**
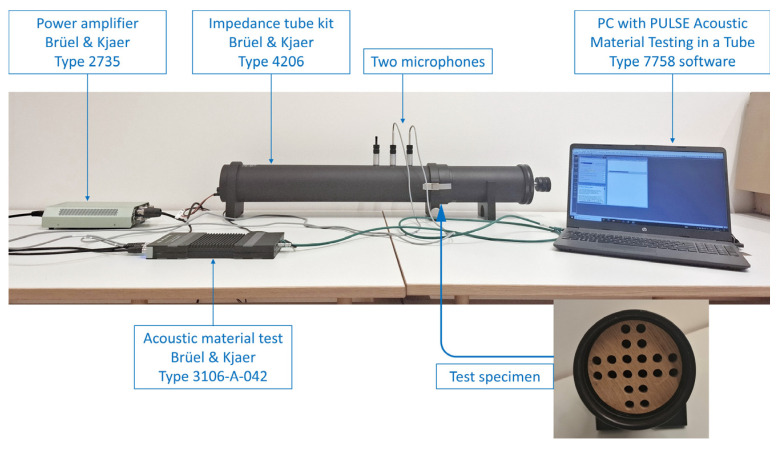
Measurement setup for determining the normal-incidence sound absorption coefficient, consisting of the Brüel & Kjær two-microphone impedance tube system BK Type 4206 and associated instrumentation.

The sound absorption coefficient of the specimens was measured using a two-microphone impedance tube Brüel & Kjær, Type 4206 with PULSE Acoustic Material Testing (Brüel & Kjær Sound &Vibration Measurement A/S, Nærum, Denmark) in a tube Type 7758 software (Pulse LabShop Version 25.01.0.255—2022-04-21, Brüel & Kjær Sound &Vibration Measurement A/S, Nærum, Denmark), following the transfer function method specified in ISO 10534-2 [1]. Panels made from four wood species: fir wood (*Abies alba*), pine wood (*Pinus sylvestris*), pedunculate oak (*Quercus robur*), and sessile oak (*Quercus petraea*) were tested at three thicknesses: 11 mm, 18 mm, and 25 mm, with perforation ratios of 0%, 10%, and 20%. These parameters were selected to represent the range commonly used in architectural applications [7,8]. A schematic representation of the measurement setup is shown in Figure 4. The loudspeaker is mounted at one end of the tube and generates stationary plane acoustic waves that propagate along the tube axis. The test specimen is placed flush at the opposite end, with its front surface perpendicular to the direction of propagation.

As the incident acoustic wave reaches the specimen surface, part of the acoustic energy is absorbed, while the remaining portion is reflected toward the source. The superposition of the forward- and backward-traveling waves creates a standing wave pattern inside the tube. The acoustic pressures at two fixed microphone positions are recorded, and the complex transfer function between them is obtained using a digital frequency analyzer. From this transfer function, the complex reflection coefficient at the specimen surface is determined, and the normal-incidence sound absorption coefficient is calculated according to ISO 10534-2.

All measurements were performed under steady-state excitation conditions. Before each measurement, the specimen was carefully positioned so that it was centered and in full contact with the inner wall of the tube, ensuring no air gaps were present between the specimen and the tube. This prevented acoustic leakage and ensured repeatability and accuracy of the measured sound absorption coefficient.

As described in Section 2, the density of the specimens was determined prior to sound absorption measurements. The results of these measurements are presented in Table 1.

The measured density values show a clear and consistent distinction among the examined wood species, with Sessile Oak and Pedunculate Oak exhibiting the highest densities, followed by Pine Wood and Fir Wood. This hierarchy aligns with well-documented differences in anatomical structure and cell wall composition, particularly the proportion of latewood, vessel arrangement, and fiber wall thickness characteristic of hardwoods and softwoods [9,10,11]. Across all species, the degree of perforation caused only minor reductions in density, as expected due to the physical removal of material. However, this effect remained comparatively small, indicating that the material’s intrinsic cellular architecture continues to dominate mass distribution even after perforation. Similarly, variation in specimen thickness did not produce any systematic trend, confirming that density is fundamentally a material property rather than a function of specimen geometry [9,12].

The standard deviations observed in the measurements were generally low, typically corresponding to only a small fraction of the mean values. This suggests good measurement repeatability and limited internal heterogeneity at the scale of the tested specimens. Slightly elevated standard deviations in a few cases are consistent with the known natural variability of wood microstructure, influenced by factors such as growth rate, ring orientation, and localized resin or vessel distribution, rather than by experimental uncertainty [2,3]. Overall, these findings demonstrate that wood species is the dominant factor influencing density, while perforation and specimen thickness introduce only secondary and comparatively minor effects [13].

All impedance-tube measurements were repeated seven times for each wood specimen. The arithmetic mean of these seven repetitions was used in the subsequent analysis. Because the differences between the minimum and maximum measured values were small and the standard deviations were low, the measurement results can be considered statistically stable, and the chosen number of repetitions is sufficient for this study. As an illustration, the distribution of repeated measurements is shown in Table 2 for three representative specimens: a pine wood panel with a thickness of 18 mm and a perforation ratio of 20%, a pedunculate oak panel with a thickness of 25 mm and a perforation ratio of 20%, and a fir wood panel with a thickness of 11 mm without perforations.

### 3.1. Absorption Coefficient as a Function of Material Density

Wood density plays a fundamental role in determining a panel’s acoustic behavior by influencing microstructural properties such as porosity, tortuosity and airflow resistivity. Lower-density woods typically have higher porosity and lower airflow resistivity, which enhance viscous and thermal dissipation and increase mid- to high-frequency absorption. However, extremely low densities may result in insufficient energy dissipation at low frequencies [14].

Existing studies have shown a linear increase in specific acoustic impedance with density, with lower-density species generally exhibiting higher absorption, although peak absorption may vary between species due to anisotropy [9]. Research has also indicated that grain orientation, even within the same species, can significantly influence absorption by altering effective porosity, with more porous orientations yielding higher Noise Reduction Coefficients (NRC) [10].

Investigations on engineered wood products have confirmed a strong relationship between density, porosity, and the absorption coefficient. Results for wood fiberboards have demonstrated a high correlation (R^2^ ≈ 0.99) between porosity and NRC across different densities [11], while studies on wood-waste panels have shown that reduced pressing pressure (and thus lower density) increases porosity and can improve absorption at approximately 0.8 at 3.2 kHz [12]. Other work on wood-chip absorbers has found that there is an optimal density range in which moderate densification improves low-frequency absorption, whereas excessive densification reduces porosity and degrades performance [15]. Furthermore, research on thermally modified hardwoods has shown that modifications affecting porosity and airflow resistivity can lead to measurable shifts in the absorption spectrum [16].

**Figure 5:**
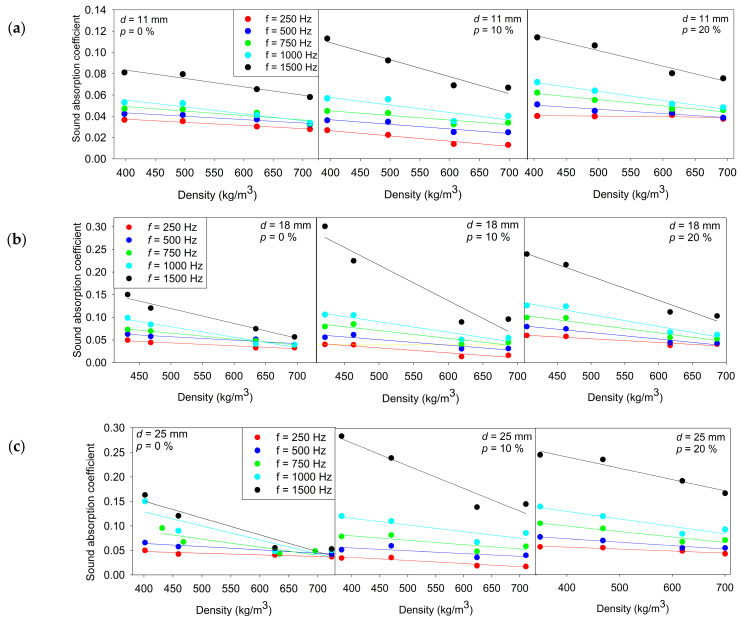
Relationship between the sound absorption coefficient and density for wood of different thicknesses: (**a**) 11 mm; (**b**) 18 mm; (**c**) 25 mm and perforation ratios (0%, 10%, and 20%) at 250 Hz, 500 Hz, 750 Hz, 1000 Hz, and 1500 Hz. Solid lines represent the corresponding linear regression fits for each measurement condition.

The measured absorption coefficients show a clear and systematic frequency dependence, with absorption increasing across the range from 250 Hz to 1500 Hz for all specimen configurations, as shown in Figure 5. Differences between perforation levels and thicknesses are relatively small at 250 Hz, where absorption values remain low regardless of geometry. However, above approximately 750 Hz, both thickness and perforation begin to play a significant role. The 18 mm and 25 mm specimens consistently exhibit higher absorption than the corresponding 11 mm specimens, indicating that increased material thickness enhances sound energy dissipation in the mid- and high-frequency regions. Similarly, perforated specimens generally show higher absorption than non-perforated specimens at 750 Hz and above, with the 10% and 20% perforation levels resulting in comparable improvements. These results demonstrate that thickness primarily influences baseline absorption capacity, while perforation effectively enhances absorption performance in the higher frequency range.

The linear regression analysis (5)

α(ρ) = a · ρ + b(5)

with α representing the sound absorption coefficient and ρ representing the specimen density shows that the slope a is negative under all tested conditions, across all thicknesses (11 mm, 18 mm, 25 mm), perforation levels (0%, 10%, 20%), and frequencies (250–1500 Hz). This consistently indicates that the sound absorption coefficient decreases as density increases. Furthermore, the magnitude of the slope |a| generally increases with frequency, meaning that the influence of density on absorption becomes more pronounced at higher frequencies. For example, for the 18 mm non-perforated specimens, |a| increases from approximately 6.5 × 10^−5^ at 250 Hz to 3 × 10^−4^ at 1500 Hz, with similar frequency dependent strengthening also observed for the 11 mm and 25 mm specimens. The intercept b shows a monotonic increase with frequency in all configurations, reflecting a higher baseline absorption level at mid-to-high frequencies regardless of density. For instance, for the 11 mm, 0% perforation series, b increases from 0.049 at 250 Hz to 0.114 at 1500 Hz, and comparable upward trends are observed for all perforation levels and thicknesses.

The effect of perforation is frequency dependent. At 1000–1500 Hz, perforated specimens often exhibit higher intercept values b compared to non-perforated ones, whereas the maximum b at 1500 Hz frequently occurs at 10% perforation rather than 20%, as seen for both 18 mm (0.284/0.607/0.455 for 0/10/20%) and 25 mm specimens (0.288/0.456/0.334). This indicates that increasing perforation from 10% to 20% does not uniformly improve absorption at higher frequencies.

The coefficient of determination R^2^ confirms the suitability of the linear model in most cases. Across 500–1500 Hz, R^2^ values are predominantly high, commonly ≥0.85, demonstrating a strong linear relationship between density and absorption in this frequency range. The lowest R^2^ values occur at 250 Hz, particularly for the 11 mm/20% perforation condition (R^2^ = 0.236), where density has limited predictive relevance. In contrast, at 1500 Hz, R^2^ values are consistently high across all thicknesses and perforation levels (e.g., for 18 mm: 0.969/0.914/0.969 for 0/10/20%).

Overall, the results clearly demonstrate a stable negative dependence of absorption on density, a systematic increase in baseline absorption with frequency, and strong model validity, high R^2^, in the 500–1500 Hz range, with expected reduced correlation at 250 Hz. These findings show that density is a reliable parameter for predicting absorption behavior in the mid-to-high frequency range. All linear regression coefficient values are presented in Table 3.

### 3.2. Absorption Coefficient as a Function of Material Thickness

Panel thickness significantly affects acoustic absorption through two main mechanisms. In porous or loose materials, increased thickness lowers the frequency at which quarter-wavelength resonance occurs, thereby improving low-frequency absorption. In perforated systems, thickness changes the effective neck length of the resonator, which affects both the resonance frequency and viscous dissipation.

Existing studies have shown that increasing the thickness of wood-chip absorbers from 30 to 50 mm enhances the absorption coefficient and shifts the peak toward lower frequencies, with similar benefits achieved by adding a thin air cavity behind the material [15]. Other research on bark mats with thicknesses from 20 to 100 mm and densities around 120–160 kg/m^3^ has reported consistent increases in absorption and NRC across different species [17]. In contrast, investigations of solid wood boards in the 20–40 mm range have found that thinner specimens can sometimes exhibit higher absorption in the 250–2000 Hz range. This has been attributed to the low internal damping and high reflectivity of solid wood, where additional mass does not necessarily improve dissipation [18].

Further studies on perforated MDF panels indicate that greater thickness improves neck-related dissipation, increases the maximum absorption coefficient, and lowers the resonance frequency, underscoring the importance of thickness in resonant absorber design [5].

**Figure 6:**
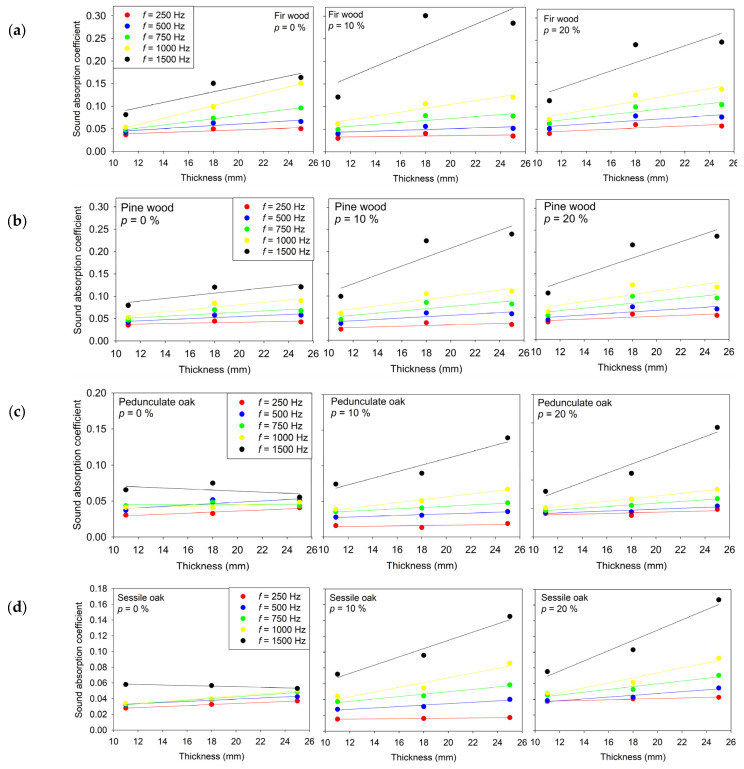
Relationship between the sound absorption coefficient and thickness for wood specimens: (**a**) Fir wood; (**b**) Pine wood; (**c**) Pedunculate oak; (**d**) Sessile oak with perforation ratios of 0%, 10%, and 20%, measured at 250 Hz, 500 Hz, 750 Hz, 1000 Hz, and 1500 Hz. Solid lines represent the corresponding linear regression fits for each measurement condition.

The measured sound absorption coefficients show clear frequency-dependent and thickness-dependent behavior across all examined wood species and perforation ratios, as shown in Figure 6. For all specimens, increasing specimen thickness generally led to higher absorption coefficients, reflected in the positive slope values of the linear fits at the most of tested frequencies.

This trend is consistent with the expected increase in internal friction and energy dissipation within thicker, porous or fibrous structures.

In this case, the linear regression is expressed by (6), where α denotes the sound absorption coefficient and d represents the sample thickness.

α(d) = a · d + b(6)

For fir wood, the linear models show a moderate correlation between thickness and absorption at lower frequencies (250–500 Hz), with R^2^ values typically below 0.80. At mid frequencies (750–1000 Hz) the correlation becomes substantially stronger, reaching R^2^ ≈ 0.99 at 750 and 1000 Hz. This indicates that thickness plays a significantly more pronounced role in absorption in the mid-frequency range. At higher frequencies (1500 Hz), the correlation weakens again, suggesting that absorption mechanisms become less thickness-driven at that range.

For pine wood, the slopes remain positive across nearly all frequencies and perforation ratios, confirming a systematic increase in absorption with thickness. However, the correlation strength (R^2^ ≈ 0.50–0.86) is generally lower compared to fir and oak at corresponding frequencies. This suggests that although the trend is present, the variability of absorption behavior with thickness is higher, which may be associated with greater heterogeneity in pine wood microstructure.

In pedunculate oak, a similar frequency dependent behavior is observed, though with greater variation across perforation ratios. At 500–1000 Hz, particularly for perforated specimens, very high linearity is recorded (R^2^ > 0.95), indicating a strong and consistent relationship between thickness and absorption. However, at 250 Hz and 1500 Hz, R^2^ values decrease, and in some cases the fitted slopes are near zero or slightly negative, suggesting that thickness alone does not significantly influence absorption in those frequency bands for this wood type. This indicates that factors such as intrinsic pore distribution and surface microstructure may have a greater influence in these cases.

Sessile oaks show the most consistent linear relationship among the tested species. High R^2^ values (typically >0.93 across all frequencies and perforation ratios) indicate that thickness is a dominant parameter influencing absorption behavior in this wood. The slope values increase gradually with frequency, reflecting increased dissipation at higher acoustic excitation levels. Even when the interceptive values vary (positive or near-zero), the thickness-to-absorption dependency remains strong and systematic.

Overall, the results shown in Table 4 confirm that wood thickness is a meaningful design parameter for controlling acoustic absorption, particularly in the mid-frequency range (750–1000 Hz), where the linear dependence is strongest across most species. The observed differences between species and perforation ratios indicate that microstructural characteristics and porosity interact with thickness effects, influencing the efficiency of energy dissipation mechanisms. These findings are relevant for the engineering design of wood-based acoustic panels where tailored absorption performance is required.

### 3.3. Absorption Coefficient as a Function of Perforation Ratio

Perforation parameters—including perforation ratio, hole diameter, panel thickness and cavity depth—play a decisive role in determining the Helmholtz resonance behavior of perforated wooden panels. These factors jointly influence both the magnitude and frequency of peak absorption.

Existing research has shown that reducing hole diameter while maintaining a constant perforation ratio can increase viscous losses, resulting in higher absorption and a slight shift in resonance frequency [4]. Other studies report that increasing the perforation ratio typically reduces the maximum absorption coefficient and shifts the resonance frequency to higher values, while increasing panel thickness can raise the maximum absorption coefficient and lower the resonance frequency [5].

Further work demonstrates that broadband, high-performance absorption can be achieved by optimizing irregular perforation patterns and combining them with a porous backing, with the overall depth carefully designed to target a wide frequency range [15]. Reviews of perforated acoustic systems confirm general trends, including that higher perforation ratios increase openness and raise resonance frequency, while smaller hole diameters and greater panel thickness increase viscous losses and lower resonance frequency; cavity depth also significantly affects bandwidth and absorption magnitude [16]. In composite absorbers, combining moderate density and thickness with an optimized open area yields broad and high absorption across speech frequencies [11].

**Figure 7:**
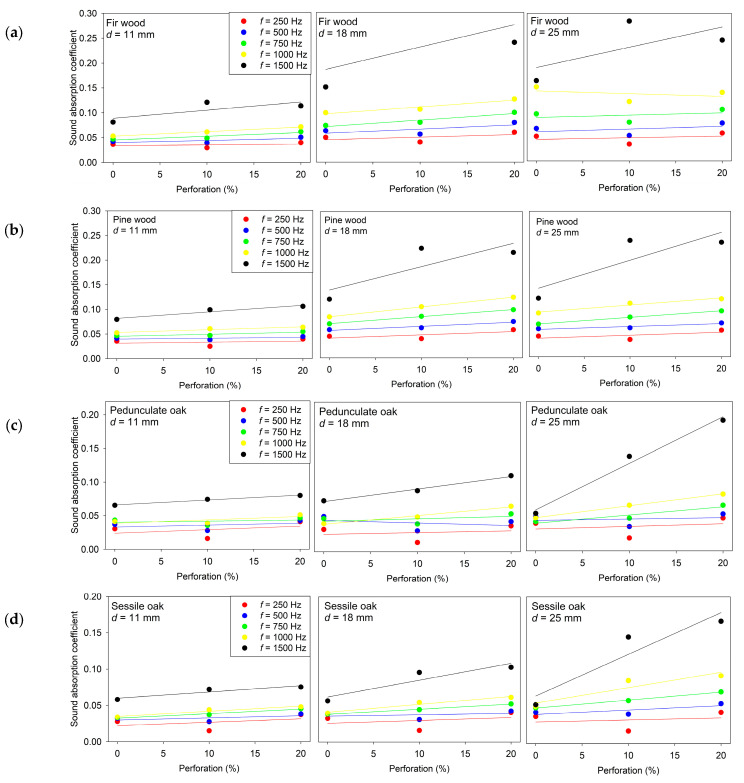
Sound absorption coefficient α as a function of perforation ratio for wooden specimens of different thicknesses measured at 250 Hz, 500 Hz, 750 Hz, 1000 Hz, and 1500 Hz: (**a**) Fir wood; (**b**) Pine wood; (**c**) Pedunculate oak; (**d**) Sessile oak.

The measurement results demonstrate a clear and systematic influence of perforation ratio on the sound absorption performance of wooden specimens, Figure 7. For all examined wood species and thicknesses, the absorption coefficient generally increases with higher perforation percentages, particularly in the mid- and high-frequency range (750–1500 Hz), where the interaction between the perforation pattern and viscous–thermal boundary layer effects become more pronounced. At lower frequencies (250–500 Hz), the influence of perforation is less significant, and the absorption remains comparatively low and less sensitive to geometric modification. Furthermore, the results show that the magnitude of absorption increase varies among different wood species and thicknesses, indicating that both material microstructure and specimen geometry contribute to the acoustic behavior in addition to perforation.

The linear regression coefficients in Table 5 quantify the relationship between perforation ratio and absorption coefficient for each wood type, specimen thickness, and frequency. The relationship between those variables is given by (7), where α denotes the sound absorption coefficient and p represents the perforation percentage.

α(p) = a · p + b(7)

The slope values are mostly positive across conditions, confirming that increasing in perforation ratio increases absorption. This trend is especially consistent at mid and higher frequencies (750–1500 Hz), where the slopes are higher and the coefficients of determination (R^2^) approach unity for several configurations. This indicates that, in these ranges, perforation serves as a dominant tuning parameter, and absorption behavior can be reliably approximated with a simple linear model.

In contrast, at lower frequencies (250–500 Hz), the slopes are smaller and the R^2^ values are generally low, indicating weak linear dependence and suggesting that the acoustic mechanism in this range is governed primarily by material stiffness and panel mass rather than perforation-induced dissipation. The results also show that the effect of perforation and the goodness of fit depend on specimen thickness: thicker specimens in several cases exhibit more pronounced and more linear perforation-dependent absorption trends at higher frequencies, while thinner specimens display less consistent sensitivity. Additionally, differences among wood species are evident; configurations with higher R^2^ values correspond to material–geometry combinations where viscous losses inside the perforations and surface pore interaction are more strongly coupled to the acoustic field.

Overall, the regression analysis confirms that perforation ratio can be considered a controllable and predictable parameter for tuning absorption performance in the mid–high frequency region, while its influence at low frequencies is limited. These findings support the use of perforated wooden elements in architectural acoustic treatments where frequency-selective absorption is desirable, particularly in spaces requiring controlled mid- and high-frequency damping without excessive low-frequency attenuation.

In addition to forward prediction, where the absorption coefficient is estimated from known material parameters, the regression relationships given by Equations (5)–(7) can also be used inversely to support practical design decisions. In many architectural applications, the design problem is naturally formulated in reverse: a target normal-incidence sound absorption coefficient (or a target range of values at selected frequency bands) is specified first, and the designer must then identify suitable combinations of wood species density, panel thickness, and perforation ratio that achieve this target under structural and visual constraints.

Since Equations (5)–(7) are linear functions of the basic parameters, they can be rearranged algebraically to solve for an unknown parameter when the others are fixed. For example, if the wood species (and thus density range) is preselected for aesthetic or availability reasons, and panel thickness is constrained by maximum allowable surface mass or construction depth, the perforation ratio required to reach a desired absorption level can be obtained directly from the corresponding regression equation. Conversely, if the perforation ratio is limited by mechanical or fabrication constraints, the same equations can be used to determine the minimum panel thickness or suitable density range needed to compensate and still meet the acoustic target.

This inverse use is particularly useful in early design stages, where rapid exploration of feasible combinations is needed and detailed numerical simulations are not yet justified.

## 4. Discussion

The results of this study provide a systematic understanding of how wood density, panel thickness, and perforation ratio affect the normal-incidence sound absorption coefficient of wooden panels.

The negative correlation between density and absorption matches the expectation that lower-density wood generally has higher internal porosity and lower airflow resistivity, which facilitates energy dissipation through viscous friction. Fir wood and pine wood, representing the lower-density species in this study, consistently exhibited higher absorption, especially at frequencies above 750 Hz. In contrast, pedunculate and sessile oak specimens showed lower absorption values, reflecting their denser, more compact cell structures. These findings are consistent with previous research on natural wood absorbers [4,5,19] and confirm that density is a primary predictor of mid-frequency absorption performance. However, at low frequencies (≤250 Hz) density had minimal influence.

Thickness had a strong effect on absorption particularly in the 750–1000 Hz range. Thicker specimens provided greater depth for internal wave dissipation, resulting in higher absorption coefficients. This is directly relevant to interior architectural design, where thickness is often limited by weight, substructure, or visual considerations. The present data offer designers’ quantitative evidence to support compensating for reduced panel thickness by adjusting density or perforation to maintain acoustic performance.

Perforation had the most pronounced effect at higher frequencies (≥750 Hz). This frequency-dependent behavior aligns with classic Helmholtz resonance-based descriptions of perforated absorbers [20]. However, the finding that 20% perforation does not necessarily yield higher absorption than 10% is particularly significant. This suggests that there is a practical optimum range of perforation, beyond which the surface becomes too acoustically transparent, reducing dissipation efficiency. This finding suports optimization-focused studies on perforated acoustic wood panels [4,19,21] and is highly relevant for practitioners, as it shows that simply increasing perforation percentage is not a reliable strategy for improving absorption [7].

A key contribution of this work is demonstrating that the absorption coefficient can be directly predicted or inversely determined from desired acoustic performance targets using simple linear functions of density, thickness, and perforation ratio. This reversibility is valuable in real architectural workflows where acoustic consultants must work within predefined visual, cost, or installation constraints. The linear models developed here are analytically transparent, require minimal computational resources, and are easily implemented in spreadsheets, BIM objects, and acoustic planning software. Such transparency is important for collaborative decision-making between architects and acoustic engineers [22].

From a modeling perspective, only first-order linear terms were retained in the regression models. This choice follows the general principle of parsimony [23], that among alternative models with similar explanatory power, the model with fewer parameters is preferred because it is typically more stable and more generalizable to new data [24]. High-order polynomial models and regressions with many interaction terms can provide an excellent fit to a given dataset but are more prone to overfitting, especially when the number of fitted coefficients becomes comparable to the number of available observations [25]. For the experimental dataset collected in this study, simple linear relationships between density, thickness, perforation ratio and the sound absorption coefficient were therefore considered more appropriate than more complex functional forms. Future work could extend the present analysis by exploring multiple regression models with interaction and nonlinear terms once larger datasets become available; such models might capture more subtle dependencies while still being constrained by the same parsimony considerations emphasized.

It should be noted that, for some parameter combinations, the coefficient R^2^ of the fitted models is relatively low. This behavior is observed mainly at the lower frequency bands, where the normal-incidence absorption coefficient shows a more irregular variation with respect to density, thickness, and perforation ratio. At these frequencies, the simple linear functions do not fully follow the detailed point-by-point structure of the measured data, resulting in larger residual scatter. This limitation is acknowledged, as linear regression was adopted as a first-order approximation to provide a relatively simple tool for engineering use. In practical design, panel thickness and wood density are typically chosen first (for example under structural load constraints), and perforation parameters are then adjusted for fine-tuning the desired frequency-dependent absorption. For this purpose, compact linear trends that highlight the dominant influence of each parameter were considered more useful than more complex models that might achieve higher accuracy at the cost of transparency and ease of application.

Furthermore, it can be observed that for some specimens, the influence of panel thickness on the sound absorption coefficient is weak or even slightly non-monotonic at low frequencies. This behavior is related to the fact that, in this frequency range, the panel thickness is very small compared to the acoustic wavelength, so the stiff wooden panels backed by a rigid termination act predominantly as reflective surfaces, and moderate changes in thickness have only a limited effect on normal-incidence absorption. At mid and higher frequencies, where the acoustic wavelength becomes comparable to the panel dimensions and the panels are more acoustically “active,” the effective path length and internal damping become more important, and the positive influence of thickness on sound absorption becomes much more pronounced.

Future research should focus on extending the practical usability and integration of the presented regression-based model into architectural and engineering workflows. Such integration would enable architects and acoustic consultants to specify a target absorption coefficient, while the tool automatically identifies feasible combinations of wood species density, thickness, and perforation ratio that meet those criteria.

Additionally, a digital material library could be developed based on the measured dataset, with each parameter combination accompanied by predicted absorption characteristics. This library could be a searchable database or interactive configurator, enabling manufacturers, interior designers, and engineers to efficiently compare alternative solutions. The library could be expanded over time through collaboration with industry partners, allowing the inclusion of additional wood species, finishes, and construction methods.

The prediction framework can also be adapted into standardized selection charts or design tables, similar to mechanical engineering material charts. For example, diagrams showing regions of high expected absorption for different density–thickness–perforation combinations would provide immediate guidance when choosing materials in early design phases. These charts would be especially useful in practical contexts where rapid, visually supported material decisions are required.

Finally, the findings of this study can inform the development of design guidelines and best-practice recommendations for selecting wooden acoustic panels in various building typologies. For example, based on the regression results, moderate perforation ratios and mid-range thicknesses may be recommended for classrooms and office environments, while density becomes a more critical factor in auditoria where large surface areas affect overall mass loading. Such guidelines would translate the analytical results into practical, decision-ready instructions for the building industry.

## 5. Conclusions

This study presents an empirical approach for estimating and selecting wooden acoustic panels by relating the normal-incidence absorption coefficient to three measurable physical parameters: density, panel thickness, and perforation ratio. The model is based on impedance tube measurements performed on four commonly used wood species at thicknesses of 11, 18, and 25 mm, with perforation levels of 0%, 10%, and 20%. The resulting regression functions express the absorption coefficient as a first-order relationship with each examined parameter.

The measurements confirm that density consistently influences acoustic behavior: lower-density species such as fir and pine exhibit higher absorption magnitudes in the mid- and high-frequency range, particularly above 750 Hz. This corresponds to their greater internal porosity and lower airflow resistivity. Panel thickness is also a relevant tuning parameter, with increased thickness generally improving absorption and providing clearer gains in the 750–1000 Hz range. Perforation effectively shapes the absorption curve, although increasing the perforation ratio beyond approximately 10% does not uniformly improve performance at higher frequencies. This indicates that, for practical panel design, perforation should be considered as an adjustable parameter with an optimal range rather than a quantity to be increased without limit [26].

A notable outcome of this work is that the derived linear relationships can be used in both directions: to estimate expected absorption based on known material parameters and, inversely, to determine which combination of density, thickness, and perforation is required to achieve a specified absorption level at selected frequencies. This reversibility makes the method suitable for early-stage design, where surface finish, mass constraints, and visual requirements are often defined before detailed acoustic simulation is completed.

In summary, this study presents a model that enables estimation or optimization of acoustic panel parameters to meet predefined performance goals. It supports both the forward and inverse problem, making it a valuable tool in architectural acoustics, interior design, and structural integration. The resulting functions are simple enough to be embedded in design tools or spreadsheets, yet accurate enough to inform early-stage decision-making. This approach is especially valuable in collaborative projects involving architects, acoustic consultants, and structural engineers where trade-offs between mass, thickness, and acoustic performance must be carefully managed.

Future work should focus on integrating these relationships into digital design tools to support faster material selection in architectural and interior acoustic applications.

## Figures and Tables

**Table 1:** Measured density values and the corresponding standard deviations of the density for the prepared wood specimens with different perforation ratios and thicknesses.

Wood Specimen	Perforation (%)	Thickness (mm)	Density (kg/m^3^)	Standard Deviation (kg/m^3^)	Moisture Content (%)
Fir wood	0	11	397.5935	9.278	9.3
		18	431.4366	9.0738	8.7
		25	401.7588	7.9672	8.6
	10	11	394.5184	12.4914	9.1
		18	422.0335	4.244	9.4
		25	381.7811	5.3559	8.6
	20	11	404.9653	5.118	9.2
		18	403.9124	3.8483	10.0
		25	349.1507	2.8985	9.1
Pine wood	0	11	496.5075	18.4304	8.6
		18	468.0661	17.6988	9.7
		25	459.4968	19.6747	9.7
	10	11	496.5151	6.5414	8.8
		18	463.3562	3.7254	8.5
		25	470.5072	18.2617	9.3
	20	11	494.1131	6.6058	8.5
		18	462.8892	3.6907	8.7
		25	468.2803	12.5921	8.9
Pedunculate oak	0	11	622.1249	16.9325	9.2
		18	634.2652	6.0915	9.4
		25	626.0737	20.2281	8.0
	10	11	606.7254	27.41	8.7
		18	619.1451	13.5896	8.8
		25	623.5055	18.7268	8.6
	20	11	614.0706	8.5249	9.8
		18	616.9498	13.0469	8.0
		25	619.0446	19.5393	8.6
Sessile oak	0	11	712.1723	16.8037	8.9
		18	695.6069	18.8408	9.3
		25	723.9248	16.3601	8.4
	10	11	698.0474	16.5236	8.7
		18	686.6924	8.8618	10.0
		25	710.8469	19.244	8.6
	20	11	693.3991	18.4744	8.6
		18	686.4827	7.5413	8.3
		25	700.2972	13.7654	8.1

**Table 2:** Descriptive statistics of repeated measurements for selected wood specimens.

Wood Specimen	f, Hz	Mean	St. Dev.	Min.	Max
Fir wood	250	0.0273	2.91 × 10^−4^	0.0267	0.0275
	500	0.0432	2.65 × 10^−4^	0.0429	0.0435
	750	0.0581	6.05 × 10^−4^	0.0574	0.0592
	1000	0.0656	5.82 × 10^−4^	0.0646	0.0663
	1250	0.0760	1.04 × 10^−3^	0.0748	0.0771
	1500	0.0905	5.18 × 10^−4^	0.0897	0.0913
Pine wood	250	0.0465	1.99 × 10^−3^	0.0450	0.0506
	500	0.0700	2.87 × 10^−3^	0.0682	0.0753
	750	0.0942	2.87 × 10^−3^	0.0922	0.0984
	1000	0.1238	5.12 × 10^−4^	0.1231	0.1244
	1250	0.1564	2.77 × 10^−3^	0.1540	0.1613
	1500	0.2028	1.84 × 10^−3^	0.2009	0.2060
Pedenculate oak	250	0.0345	8.67 × 10^−4^	0.0326	0.0351
	500	0.0515	3.42 × 10^−4^	0.0512	0.0522
	750	0.0715	4.99 × 10^−4^	0.0704	0.0721
	1000	0.0966	6.82 × 10^−4^	0.0954	0.0972
	1250	0.1303	2.34 × 10^−3^	0.1263	0.1329
	1500	0.1825	5.53 × 10^−4^	0.1813	0.1831

**Table 3:** Linear fit parameters describing the relationship between the sound absorption coefficient and density for wood specimens of different thicknesses and perforation ratios measured at 250 Hz, 500 Hz, 750 Hz, 1000 Hz, and 1500 Hz.

d (mm)	f (Hz)	*p* = 0%			*p* = 10%			*p* = 20%		
		a	b	R^2^	a	b	R^2^	a	b	R^2^
11	250	−2.95 × 10^−5^	0.049	0.967	−5.22 × 10^−5^	0.050	0.941	−5.92 × 10^−6^	0.042	0.236
	500	−3.17 × 10^−5^	0.055	0.934	−4.57 × 10^−5^	0.058	0.873	−3.95 × 10^−5^	0.066	0.937
	750	−4.18 × 10^−5^	0.065	0.787	−4.59 × 10^−5^	0.067	0.812	−5.90 × 10^−5^	0.084	0.954
	1000	−6.46 × 10^−5^	0.081	0.930	−7.44 × 10^−5^	0.092	0.719	−8.55 × 10^−5^	0.105	0.982
	1500	−7.85 × 10^−5^	0.114	0.943	−2.00 × 10^−4^	0.184	0.936	−1.00 × 10^−4^	0.174	0.959
18	250	−6.50 × 10^−5^	0.076	0.942	−1.00 × 10^−4^	0.087	0.901	−8.22 × 10^−5^	0.093	0.889
	500	−7.58 × 10^−5^	0.095	0.885	−1.00 × 10^−4^	0.111	0.873	−1.00 × 10^−4^	0.139	0.958
	750	−0.0001	0.130	0.996	−0.0002	0.156	0.866	−0.0002	0.181	0.941
	1000	−0.0002	0.195	0.962	−0.0002	0.206	0.914	−0.0003	0.237	0.949
	1500	−0.0003	0.284	0.969	−0.0008	0.607	0.914	−0.0005	0.455	0.969
25	250	−3.38 × 10^−5^	0.061	0.823	−6.26 × 10^−5^	0.060	0.888	−4.01 × 10^−5^	0.072	0.939
	500	−6.67 × 10^−5^	0.091	0.961	−5.63 × 10^−5^	0.077	0.591	−7.08 × 10^−5^	0.101	0.951
	750	−0.0002	0.155	0.778	−8.98 × 10^−5^	0.115	0.674	−1.00 × 10^−4^	0.144	0.912
	1000	−0.0003	0.243	0.794	−0.0001	0.171	0.710	−0.0002	0.190	0.880
	1500	−0.0003	0.288	0.911	−0.0005	0.456	0.924	−0.0002	0.334	0.952

**Table 4:** Linear fit parameters describing the relationship between the sound absorption coefficient and specimen thickness for wood specimens with perforation ratios of 0%, 10%, and 20% measured at 250 Hz, 500 Hz, 750 Hz, 1000 Hz, and 1500 Hz. The table reports the slope (a), intercept (b), and coefficient of determination (R^2^) for each wood type and measurement condition.

Wood Specimen	f (Hz)	*p* = 0%			*p* = 10%			*p* = 20%		
		a	b	R^2^	a	b	R^2^	a	b	R^2^
Fir wood	250	0.0017	0.025	0.853	0.0003	0.028	0.205	0.0012	0.030	0.613
	500	0.001	0.028	0.779	0.0009	0.033	0.503	0.0019	0.035	0.680
	750	0.0035	0.009	0.992	0.0021	0.030	0.729	0.0031	0.033	0.842
	1000	0.007	−0.024	0.998	0.0042	0.019	0.921	0.0048	0.025	0.889
	1500	0.0059	0.026	0.864	0.0117	0.025	0.674	0.0094	0.030	0.782
Pine wood	250	0.0005	0.031	0.574	0.0008	0.019	0.502	0.0011	0.031	0.610
	500	0.0012	0.031	0.751	0.0015	0.025	0.678	0.0018	0.031	0.606
	750	0.0015	0.034	0.659	0.0025	0.026	0.670	0.0028	0.031	0.669
	1000	0.0027	0.026	0.865	0.0036	0.027	0.830	0.004	0.030	0.685
	1500	0.0029	0.054	0.757	0.0101	0.007	0.829	0.0093	0.019	0.861
Pedunculate oak	250	0.0009	0.029	0.681	0.0002	0.012	0.268	0.0005	0.032	0.457
	500	0.0007	0.021	0.897	0.0006	0.021	0.961	0.0008	0.032	0.827
	750	−2.21 × 10^−5^	0.045	0.260	0.0009	0.025	0.989	0.0015	0.031	0.993
	1000	0.0005	0.034	0.737	0.002	0.015	0.992	0.0023	0.025	0.999
	1500	−0.0007	0.078	0.274	0.0046	0.017	0.915	0.008	−0.016	0.940
Sessile oak	250	0.0006	0.020	0.999	0.0001	0.013	0.995	0.0004	0.033	0.976
	500	0.0007	0.024	0.971	0.0009	0.016	0.935	0.0012	0.024	0.935
	750	0.0011	0.020	0.967	0.0015	0.019	0.969	0.0018	0.024	0.938
	1000	0.0012	0.021	0.972	0.003	0.007	0.925	0.0032	0.010	0.951
	1500	−0.0004	0.062	0.935	0.0052	0.010	0.960	0.0065	−0.002	0.951

**Table 5:** Linear regression parameters describing the relationship between the sound absorption coefficient and perforation ratio for wooden specimens of different thicknesses measured at 250 Hz, 500 Hz, 750 Hz, 1000 Hz, and 1500 Hz. The table reports the slope (a), intercept (b), and coefficient of determination (R^2^) for each wood type and measurement condition.

Wood Specimen	f (Hz)	d = 11 mm			d = 18 mm			d = 25 mm		
		a	b	R^2^	a	b	R^2^	a	b	R^2^
Fir wood	250	0.0002	0.033	0.099	0.0005	0.044	0.274	0.0003	0.043	0.084
	500	0.0004	0.040	0.528	0.0008	0.057	0.478	0.0005	0.059	0.183
	750	0.0007	0.045	0.830	0.0013	0.071	0.918	0.0005	0.088	0.119
	1000	0.0009	0.052	0.994	0.0014	0.096	0.928	−0.0006	0.142	0.134
	1500	0.0016	0.089	0.590	0.0045	0.185	0.349	0.0041	0.191	0.442
Pine wood	250	0.0002	0.031	0.279	0.0007	0.040	0.498	0.0006	0.001	0.395
	500	0.0002	0.039	0.277	0.0008	0.056	0.912	0.0006	0.056	0.863
	750	0.0004	0.045	0.783	0.0014	0.070	0.998	0.0014	0.067	0.998
	1000	0.0006	0.053	0.937	0.002	0.084	0.999	0.0015	0.092	0.954
	1500	0.0013	0.081	0.931	0.0048	0.139	0.683	0.0058	0.141	0.725
Pedunculate oak	250	0.0005	0.023	0.177	0.0003	0.025	0.201	0.0004	0.032	0.064
	500	0.0003	0.033	0.145	−0.0004	0.045	0.122	0.0002	0.044	0.040
	750	0.0002	0.040	0.083	0.0004	0.044	0.227	0.0012	0.040	0.897
	1000	0.0005	0.038	0.571	0.0013	0.040	0.981	0.0018	0.048	0.994
	1500	0.0007	0.066	0.985	0.0018	0.073	0.987	0.0068	0.060	0.983
Sessile oak	250	0.0005	0.022	0.179	0.0004	0.025	0.100	0.0003	0.029	0.043
	500	0.0003	0.028	0.310	0.0002	0.035	0.100	0.0006	0.039	0.609
	750	0.0006	0.032	0.963	0.0007	0.038	0.995	0.0011	0.048	0.997
	1000	0.0007	0.034	0.942	0.0011	0.041	0.957	0.0021	0.055	0.862
	1500	0.0009	0.059	0.88	0.0023	0.062	0.862	0.0057	0.064	0.885

## Data Availability

The original contributions presented in this study are included in the article. Further inquiries can be directed to the corresponding author.
